# Restoration of Remote PPG Signal through Correspondence with Contact Sensor Signal

**DOI:** 10.3390/s21175910

**Published:** 2021-09-02

**Authors:** So-Eui Kim, Su-Gyeong Yu, Na Hye Kim, Kun Ha Suh, Eui Chul Lee

**Affiliations:** 1 Department of AI & Informatics, Graduate School, Sangmyung University, Seoul 03016, Korea; soeui291@gmail.com (S.-E.K.); tnrud7495@gmail.com (S.-G.Y.); nahelove03@gmail.com (N.H.K.); 2 R&D Team, Zena Inc., Seoul 04782, Korea; kunha.suh@zenacare.net; 3 Department of Human-Centered Artificial Intelligence, Sangmyung University, Seoul 03016, Korea

**Keywords:** photoplethysmography, support vector regression, deep learning, contact PPG, remote PPG, signal restoration

## Abstract

Photoplethysmography (PPG) is an optical measurement technique that detects changes in blood volume in the microvascular layer caused by the pressure generated by the heartbeat. To solve the inconvenience of contact PPG measurement, a remote PPG technology that can measure PPG in a non-contact way using a camera was developed. However, the remote PPG signal has a smaller pulsation component than the contact PPG signal, and its shape is blurred, so only heart rate information can be obtained. In this study, we intend to restore the remote PPG to the level of the contact PPG, to not only measure heart rate, but to also obtain morphological information. Three models were used for training: support vector regression (SVR), a simple three-layer deep learning model, and SVR + deep learning model. Cosine similarity and Pearson correlation coefficients were used to evaluate the similarity of signals before and after restoration. The cosine similarity before restoration was 0.921, and after restoration, the SVR, deep learning model, and SVR + deep learning model were 0.975, 0.975, and 0.977, respectively. The Pearson correlation coefficient was 0.778 before restoration and 0.936, 0.933, and 0.939, respectively, after restoration.

## 1. Introduction

Photoplethysmography (PPG) is an optical measurement technique that detects changes in blood volume in the microvascular layer caused by the pressure generated by the heartbeat [1]. It is usually measured in contact with the surface of the skin, such as the ear or finger. The contact measurement of PPG causes inconvenience to PPG measurers.

To solve the inconvenience of contact PPG (cPPG) measurement, a technology that can measure PPG in a non-contact manner using a camera has been developed [2,3]. Camera-based remote PPG (rPPG) measures subtle color changes in the skin areas extracted from camera images. The light reflected from the skin region detected by the camera is composed of a specular component and a diffuse component. The diffuse reflection component contains information about the changes in blood volume caused by the heartbeat. This information was used to measure the rPPG. Recently, a technology capable of measuring this even with a general webcam was developed [3]. Because rPPG is a non-contact method, it is convenient and has the advantage that it can be easily measured using a general camera device. However, the rPPG signal has a smaller pulsation component than the cPPG signal, and its shape is blurred. Therefore, in previous studies, only heart rate information could be obtained from the rPPG signal. By restoring rPPG to the cPPG level, medical information can be obtained through the dicrotic notch and dicrotic peak as well as the heart rate measurement [4,5].

A previous study on signal restoration detected a signal contaminated with motion noise using statistical parameters of the cPPG signal and then removed the contaminated portion to restore the cPPG signal [6]. This study reconstructed the signal by removing the unusable signal, rather than reconstructing the signal itself. Another study aimed to restore the signal itself. The signal was restored by adaptively removing noise from the signal by inputting the cPPG signal and the three-axis acceleration signal [7]. This method is inconvenient because the signal can be restored only by simultaneously collecting two signals using two sensors. Another study aimed to restore the cPPG signal to the level of an electrocardiogram (ECG) signal using support vector regression (SVR). This study extracted five correspondence feature points from each signal. Then, after learning using five SVRs for amplitude estimation and five SVRs for position estimation, the cPPG signal was restored to the level of the ECG signal [8].

In this study, 30 correspondence feature points were selected from each of the cPPG and rPPG signals. They were learned by providing them as input to three models: SVR, three-layer simple deep learning, and deep learning after multi-input SVR. Cosine similarity and Pearson correlation coefficients were used to evaluate the similarity of the restored signals.

This paper is structured as follows: Section 2.1 describes the need for rPPG restoration (the meaning of the cPPG signal waveform and the degradation of the rPPG signal). Section 2.2 describes the collection and preprocessing of the data used, and Section 2.3 describes the model used. Section 3 describes the experimental results, and Section 4 presents conclusions and future plans.

## 2. Materials and Methods

### 2.1. Need to Restore PPG Signal

#### 2.1.1. Information of cPPG Signal Waveform

**Figure 1:**
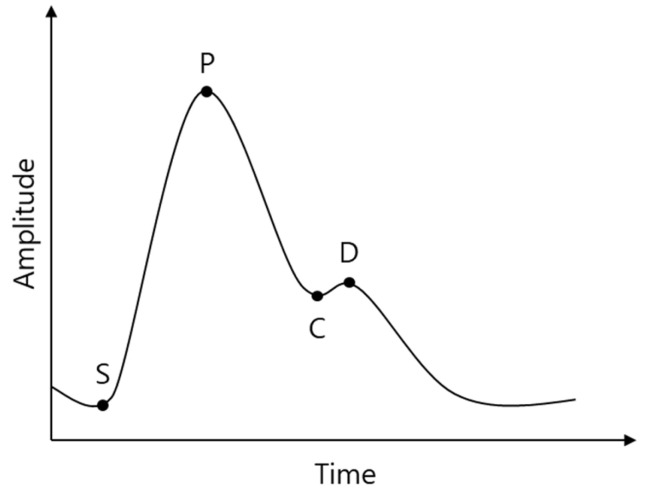
cPPG feature points: S, start point; P, systolic peak; C, dicrotic notch; D, diastolic peak.

The shape of the cPPG signal results from the contraction and relaxation of the left ventricle and wave reflections from the periphery. The cPPG has feature points as shown in Figure 1 [9].

It is generally accepted that cPPG provides important information about the cardiovascular system [1]. Several studies have been conducted on the cPPG signal waveforms [4,10,11]. Previous studies have shown that with aging, hypertension, and arteriosclerosis, the time between P and D decreases, the height of C and D increases, and D is lost. By restoring the rPPG signal to the cPPG level, it is expected that medical information such as aging, hypertension, and arteriosclerosis can be obtained with rPPG.

#### 2.1.2. rPPG Signal Degradation Model

**Figure 2:**
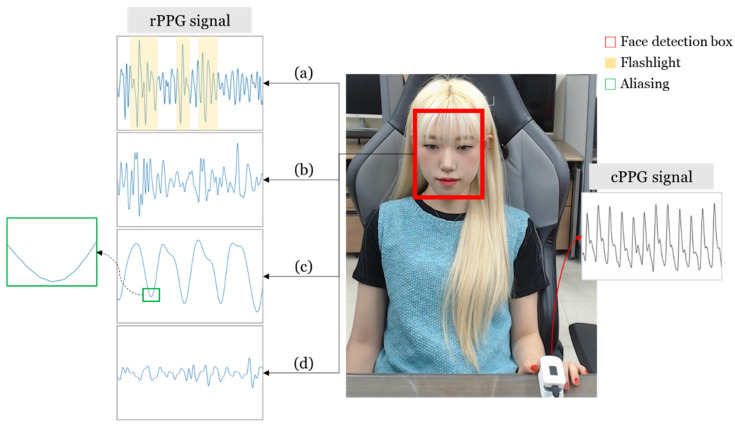
rPPG signal degradation model: (**a**) Abrupt changes in amplitude by illumination factor; (**b**) motion artifacts caused by facial movements; (**c**) aliasing due to lower sampling rate compared to cPPG; (**d**) poor signal quality due to the low sensitivity of the image sensor.

Various factors cause rPPG to degrade its signal compared with cPPG. This is shown in Figure 2.

(a) Illumination: rPPG is obtained using a camera. Therefore, it is affected by changes in illumination variation, shadows, and low luminance. In previous studies, to reduce the effect of illumination, the reflection component of illumination and the reflection component of heart rate information were separated [2,3]. This method was also used in our study. To determine the effect of illumination, we tested changes in the rPPG signal with and without a flashlight on the face.

(b) Motion: Motion noise is a problem that can easily appear in rPPG and cPPG. The cause of signal degradation due to movement may be a change in the face area or a change in the shadow of the face. Our laboratory rPPG measurement system was developed to be robust against movement. Therefore, to confirm the rPPG signal problem caused by motion, the signal was obtained by shaking the face vigorously.

(c) Sampling rate: The cPPG signal is usually obtained with a high sampling rate of more than 200. However, when rPPG is obtained with a normal camera, the FPS is 20–30, so it has a lower sampling rate than cPPG. In general, the resting heart rate of an adult is–1–1.6 beats per second, so a sampling rate of 30 is not insufficient to express a heartbeat cycle. However, compared to cPPGs with a high sampling rate, there is inevitably insufficient information, and thus signal degradation occurs. In this study, cubic interpolation was used to compensate for the aliasing problem of rPPG to some extent and to correspond to rPPG and cPPG. More details on this are provided in Section 2.3.2.

(d) Image sensor: As mentioned earlier, the rPPG was obtained through the camera. Therefore, the performance of the image sensor is affected. That is, it is affected by factors such as CCD (CMOS) sensitivity and image compression. In our lab, it was designed such that the rPPG signal could be obtained even with the webcam built into the laptop. However, the difference in the rPPG signal quality according to the performance of the image sensor is inevitable.

Among the four factors that degrade the quality of the rPPG signal, the motion artifact factor, as shown in Figure 2b, is a problem that cannot be overcome by the proposed method because the cycle of the signal is not maintained. However, factors such as Figure 2a,c,d are such that the resolution or amplitude of the shape of a single cycle signal becomes noisy while the cycle of the signal is maintained, so the quality of the signal can be improved by the proposed method.

### 2.2. Dataset

#### 2.2.1. Data Collection

For correspondence, the cPPG and rPPG signals were simultaneously obtained. Data were obtained from 10 subjects (female: 5, male: 5, age: 20 s) for 3 min each. When training, clean data should be put as input to avoid ‘garbage in, garbage out’. Therefore, only data without noise, which are difficult to remove with simple filtering, were used.

##### cPPG

**Figure 3:**
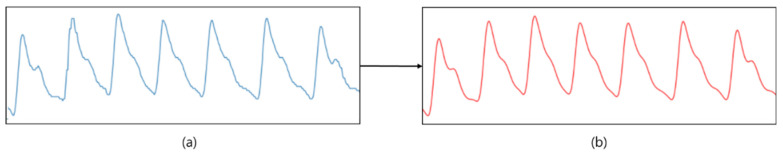
cPPG signal denoising using FIR filter: (**a**) before; (**b**) after.

The cPPG was measured using an ubpulse 360 instrument [12]. The sampling rate was set at 255 Hz. After collecting the cPPG from each subject, a finite impulse response filter (FIR), which has the advantage that the shape of the signal is well maintained even after filtering, was used for low-pass filtering of noise. The cutoff frequency, which determines the frequency band, was set to 32 Hz, and the filter coefficient was set to 40. This is an empirical value. The denoising results are shown in Figure 3.

##### rPPG

**Figure 4:**
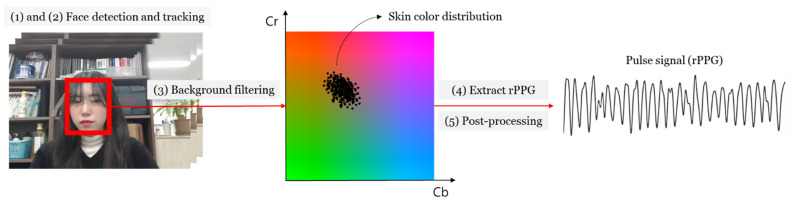
Structure of rPPG signal extraction: (**1**) Face region detection; (**2**) face region tracking using a kernelized correlation filter tracker; (**3**) extracting skin region from the face region; (**4**) rPPG signal extraction in Cb-Cr plane; (**5**) post-processing for denoising using detrending and bandpass filtering.

To obtain the rPPG signal, a video was obtained at 30 FPS on a 1080p webcam and the frame has a resolution of 640 × 480 [13]. Therefore, the sampling rate of the rPPG signal was obviously determined to be 30 Hz. The distance from the camera to the subject was specified as about 70 cm, the distance at which a face can be captured within the camera frame. There is no standardized method for extracting rPPG. In this paper, we refer to the method in [3], developed in our laboratory. In study [3], the myocardial component of the signal was enlarged to reduce the risk of distortion due to the uncertainty of the input signal. The process used to obtain rPPG in this study was as follows: (1) Detect faces in image frames. (2) Use a kernelized correlation filter (KCF) tracker to track the face. (3) Filter background pixels from rectangular face area. (4) Extract rPPG by modifying the chrominance-based method (CHROM) [2]. The CHROM collects sequential RGB images and projects them on the chromaticity plane to obtain motion-robust pulse signals. In this study, RGB images are converted into YCbCr images and the Cb and Cr signals are converted to create a single pulse signal. (5) Post-processing is performed for denoising from the signal: (i) remove trends such as breathing and (ⅱ) Butterworth bandpass filtering (42~240 bpm) to remove elements independent of cardiac activity. Figure 4 shows the structure of the rPPG extraction. A demonstration video of the collecting the rPPG based on this rPPG acquisition process can be found in [14].

#### 2.2.2. Training Data

The cPPG and rPPG signals obtained in Section 2.3.1, were split into single cycle units: cPPG used a kernel of size 300 to filter the signal by moving average and then splits the signal using the zero-crossing point of the gradient. The rPPG segmented the signal using the zero-crossing point of the gradient. The signal of the single cycle unit thus obtained was min–max normalized between 0 and 1. Thirty corresponding points of equal spacing were extracted based on the time axis of each signal. The time axis exists only for integer points. Therefore, 30 points divided into equal intervals were extracted using cubic interpolation based on the time axis of each signal.

**Figure 5:**
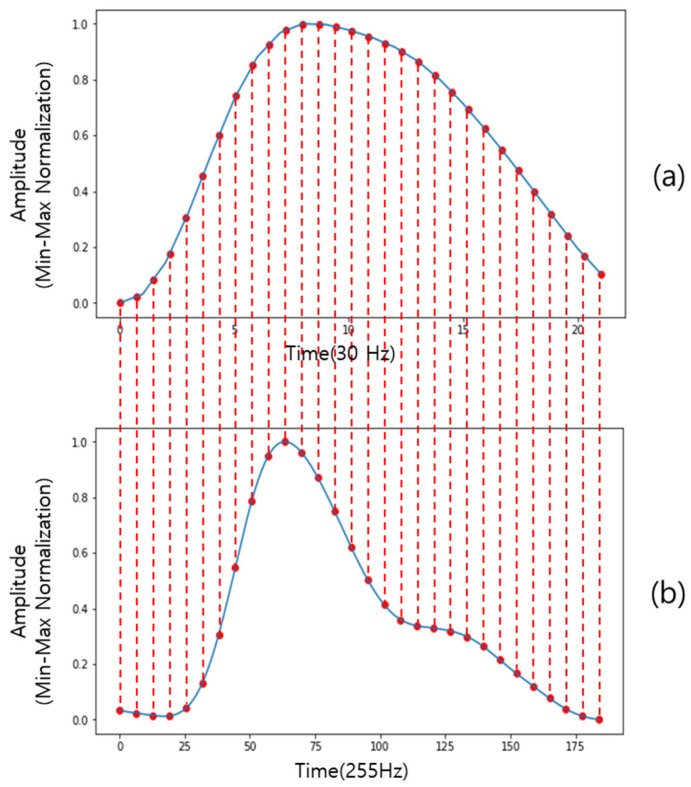
Correspondence of single cycle signal pairs: (**a**) rPPG; (**b**) cPPG.

Thus, a total of 1731 pairs of data of length 30 to be used for training were obtained. Each data pair is a correspondence. That is, there are 30 pairs of corresponding points per signal cycle. Figure 5 is an example of a single cycle signal, which is a visualization of how the signals correspond. The red dotted line represents the correspondence between the two signals.

### 2.3. Method

**Figure 6:**
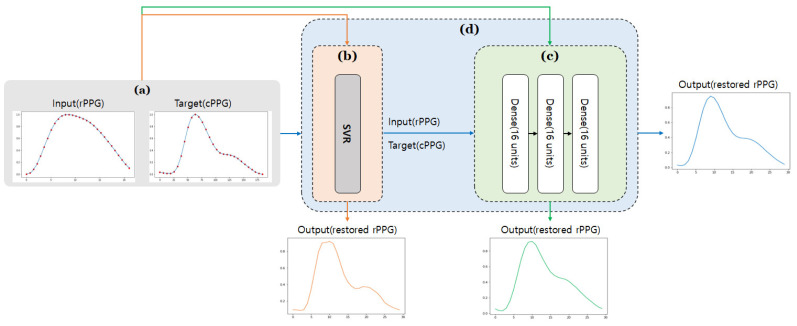
Flow chart of the proposed rPPG restoration method: (**a**) Training data through the corresponding points of rPPG and cPPG; (**b**) SVR-based restoration model (explained in Section 2.3.1); (**c**) a restoration model based on deep learning (explained in Section 2.3.2); (**d**) a restoration model using SVR and deep learning together (explained in Section 2.3.3).

The training was carried out using three models: multiple input/output SVR, a simple three-layer deep learning model, and an SVR + deep learning model. The structure of each model is shown in Figure 6, and the description of each model is as follows.

All methods were implemented in Python and run on a laptop with an Intel Core i7 (2.70 GHz) CPU and 16 GB RAM. SVR was developed using Python’s Scikit-learn library, and deep learning was developed using Python’s TensorFlow library [15,16].

#### 2.3.1. SVR

SVR is used to construct a regression equation by introducing a loss function to the representative classification algorithm SVM [17]. For SVR training, 1454 pairs of training data and 347 pairs of test data were used (each data set does not share the same subject). The error tolerance was set to 50, and a polynomial kernel function was used. Additionally, it was set to classify into quartic. The structure of the SVR model is shown in Figure 6b.

#### 2.3.2. Deep Learning

For deep learning training, 1107 pairs of training data, 347 pairs of test data, and 277 pairs of validation data were used (each data set does not share the same subject). It consisted of three dense layers, and each layer has 16 units. A dense layer is a layer in which each input unit is connected to an output unit. To initialize the weights of each layer, He initialization was used [18], and elu, which solved the dying ReLU problem while including all the advantages of the existing ReLU, was used for the activation function [19]. In addition, overfitting was prevented by the addition of an L2 weighting regulation (0.001). At the time of compilation, MAE (mean absolute error), which is often used as a regression indicator, was used for the loss function, and the optimizer used Adam, a method combining Momentum and RMSProp. To supplement the relatively small data set, cross-validation (k = 4) was performed using the training data set and the validation data set. The structure of the deep learning model is shown in Figure 6c.

#### 2.3.3. SVR + Deep Learning

The results obtained through SVR in Section 2.3.1, were used as inputs to the deep learning model in Section 2.3.2. Thus, it is expected that the advantages of SVR and deep learning can be fused. As in Section 2.3.2, cross-validation was performed. This structure can be seen in Figure 6d.

## 3. Results

Cosine similarity and Pearson correlation coefficients were used to evaluate the similarity of signals before and after restoration. Cosine similarity is the degree of similarity between two vectors, obtained using the cosine angle between the two vectors. The Pearson correlation coefficient is a numerical value that quantifies the linear correlation between two variables. It is standardized by dividing the covariance of the two variables by the product of their standard deviations. The results are shown in Table 1 (rounded to four decimal places). The cosine similarity of the signal before restoration was 0.921, and the Pearson correlation coefficient was 0.778. After restoration, significant results were obtained for all three models. Among them, the SVR + deep learning model showed the best results with a cosine similarity of 0.977 and a Pearson correlation coefficient of 0.939. The *p*-value is <0.001.

Since all data satisfies normality, a Z-test was performed for statistical verification. Table 2 shows the results of the Z-test. Through this, it was statistically verified that the results of restoring rPPG to cPPG using SVR + Deep and Deep made no significant difference from cPPG.

To judge the effectiveness of learning, the learned model was applied to the data not used in the experiment. The results are shown in Table 3 (rounded to four decimal places). It can be confirmed that significant results were obtained even with the data not used in the experiment.

**Figure 7:**
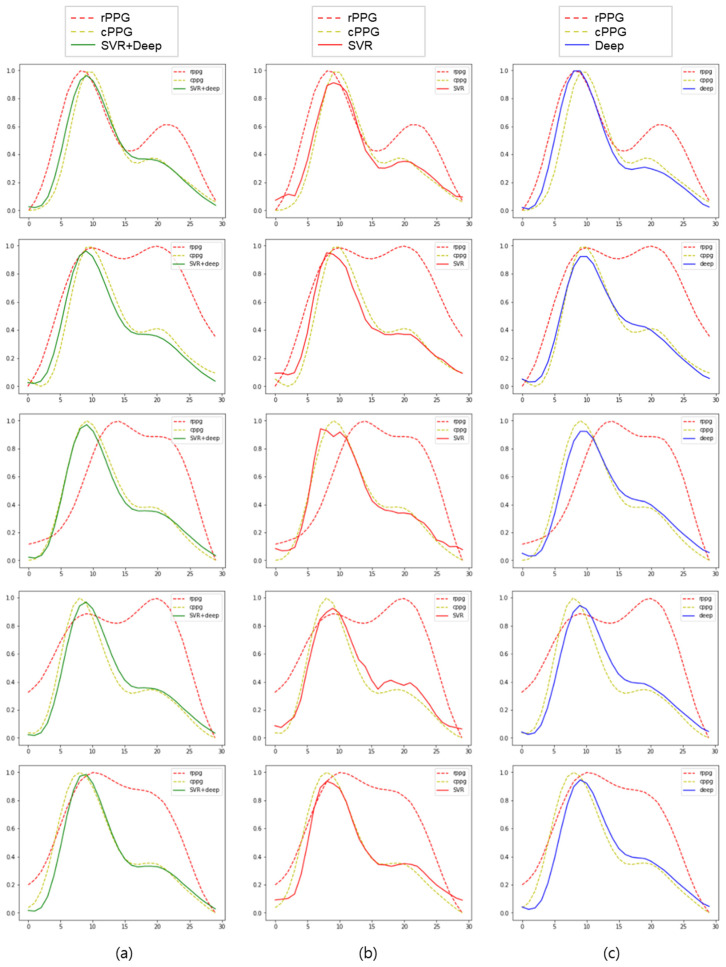
Some of the results of applying the training model to new data: (**a**–**c**) cPPG signal (yellow dotted line) and rPPG signal (red dotted line); (**a**) SVR + deep results (green solid line); (**b**) SVR results (red solid line); (**c**) deep learning results (blue solid line).

Figure 7 shows some of the learning results. Overall, it can be seen that the signal was restored to some extent. The SVR results similarly followed the trend of the cPPG signal but produced a somewhat coarser signal (c). The result of deep learning shows a smoother signal compared to the result of SVR, but there is a slight delay, and the dicrotic notch and diastolic peak (Points C and D in Figure 1) becomes unclear (d). By combining SVR and deep learning, a smooth signal was obtained, and the delay was less and clearer than that of deep learning alone (b). Thus, it can be inferred that the disadvantages of SVR and deep learning are offset, and the advantages are fused, resulting in better results.

**Figure 8:**
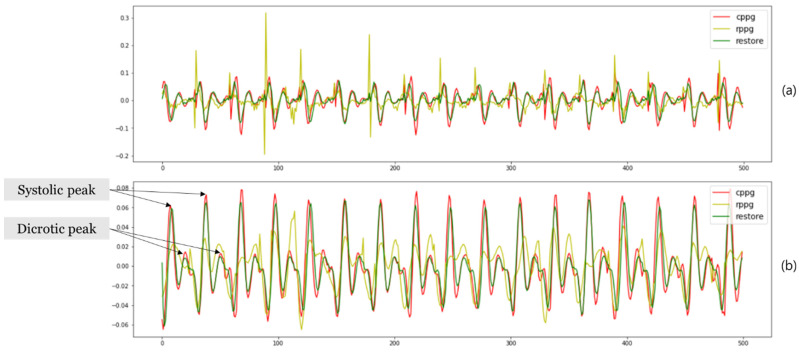
Example of second differentiation result for peak detection of cPPG, rPPG, and restored rPPG: (**a**) The result of second differentiation of cPPG, rPPG, and restored rPPG (using SVR + deep); (**b**) the result of applying and inverting the Savitzky–Golay filter in (**a**).

In Section 2.1.1, it was mentioned that the P and D (systolic peak and diastolic peak) are important in the paper. Therefore, systolic-diastolic peak-to-peak times (SD-PPTs), the interval between systolic and diastolic peaks, was measured [9]. When calculating SD-PPTs, data from three subjects of the same age were used. To obtain a clear peak, the second differentiation was performed on the cPPG signal, the rPPG signal, and the rPPG signal restored using SVR + deep. The result is shown in Figure 8a. After the second differentiation, the Savitzky–Golay filter was used to smooth the signal and then inverted. The result is shown in Figure 8b. Mean SD-PPTs of cPPG signal and rPPG signal were obtained. This is shown in Table 4. For statistical verification of the similarity of the results, a paired *t*-test was performed after confirming whether the SD-PPTs results satisfy normality. The results are shown in Table 4. Since a relatively high *p*-value was calculated for all cases, it can be seen that there is no statistically significant difference in SD-PPTs of cPPG and restored rPPG.

## 4. Conclusions

In order to improve the signal shape of rPPG signals, which were developed to solve the inconvenience of cPPG signals, a study on the restoration of the rPPG signal through correspondence with cPPG was conducted. The cPPG and rPPG signals obtained at the same time point were pretreated and then split into a single cycle unit. Then, 30 corresponding points were extracted from each signal and used as the training data. Three models were used: support vector regression (SVR), a simple three-layer deep learning model, and an SVR + deep learning model. As a result of the training, all three methods yielded significant results, and the SVR + deep learning model yielded the best results. Further, it was possible to restore the rPPG signal to the level of the cPPG signal through the experiments in this study. Using this method, medical information can be obtained with rPPG.

In the future, we will conduct a study to extract and compare various medical information from the cPPG signal and the reconstructed rPPG signal. In addition, by analyzing the morphological similarities and differences between the PPG and the ECG signals, we will proceed with a study that can infer the characteristics of the ECG signal from the rPPG signal. In addition, we will compare the restored rPPG signal and cPPG signal in terms of biomarkers such as pulse rate variability (PRV) and oxygen saturation.

## Figures and Tables

**Table 1:** Training results in terms of similarity before and after rPPG restoration by using two metrics such as cosine similarity and Pearson correlation coefficient with *p*-value.

Similarity Evaluation Index	Before Restoration	After Restoration		
		SVR + Deep	SVR	Deep Learning
Cosine similarity	0.921	0.977	0.975	0.975
Pearson correlation coefficient (*p*-value)	0.778 (4.4 × 10^−4^)	0.939 (2.4 × 10^−6^)	0.936 (1.4 × 10^−5^)	0.933 (7.6 × 10^−6^)

**Table 2:** Z-test of before and after rPPG restoration for statistical validation.

Z-Test	Before Restoration	After Restoration		
		SVR + Deep	SVR	Deep Learning
*p*-Value	0 (<0.05)	0.99 (>0.05)	0.00097 (<0.05)	0.82 (>0.05)

**Table 3:** Test results in terms of similarity before and after rPPG restoration by using two metrics such as cosine similarity and Pearson correlation coefficient with the *p*-value.

Similarity Evaluation Index	Before Restoration	After Restoration		
		SVR + Deep	SVR	Deep Learning
Cosine similarity	0.879	0.979	0.976	0.975
Pearson correlation coefficient (*p*-value)	0.637 (4.5 × 10^−3^)	0.945 (2.7 × 10^−7^)	0.937 (2.1 × 10^−6^)	0.934 (1.7 × 10^−7^)

**Table 4:** The mean SD-PPTs and paired *t*-test results of SD-PPTs of three 23-year-old subjects.

SD-PPTs		Subject 1	Subject 2	Subject 3
Mean SD-PPTs (standard deviation)	cPPG	0.274 s (±0.021 s)	0.278 s (±0.021 s)	0.256 s (±0.039 s)
	restored rPPG	0.268 s (±0.010 s)	0.269 s (±0.023 s)	0.258 s (±0.019 s)
Paired I-test (*p*-value)	restored rPPG	0.377	0.261	0.762

## Data Availability

Data cannot be shared because private information such as faces is included in the data, and an experimental consent was obtained that will only be used in this study.
